# The genetic context of *bla*_IMP_ varies among bacterial families from One Health sources

**DOI:** 10.1371/journal.pone.0327200

**Published:** 2025-07-23

**Authors:** Susan Vaughn Grooters, Dixie F. Mollenkopf, Thomas E. Wittum

**Affiliations:** Department of Veterinary Preventive Medicine, The Ohio State University, Coumbus, Ohio, United States of America; University of Florida, UNITED STATES OF AMERICA

## Abstract

The *bla*_IMP_ resistance gene encodes a metallo-beta-lactamase in bacteria, which confers reduced susceptibility or resistance to all the beta-lactams, including carbapenems which are critical for treating life-threatening infections. The dissemination of *bla*_IMP_ among various taxonomic families shows the diversity and range of horizontal gene transfer. Using short-read whole genome sequencing and bioinformatic tools, we determined the genetic motifs surrounding *bla*_IMP_ present in 32 bacterial isolates recovered from environmental sources and agriculture facilities. *bla*_IMP_ can be located extra-chromosomally on plasmids or within incomplete and complete Tn7 chromosomal structures. We identified a complete Tn7 transposon harboring the *bla*_IMP-27_ gene cassette within a class 2 integron located in chromosomal contigs of *Shewanella* spp*.* and *Providencia* spp. *Acinetobacter* spp. isolates were observed with truncated and incomplete Tn7 transposons, while conserving the class 2 integron and resistance gene cassettes. Additionally, IncQ1 plasmids carried by *Proteus* spp*., Escherichia coli,* and other *Enterobacteriaceae* spp*.* harbored class 2 integrons with *bla*_IMP-64_ and *sat*2 resistance gene cassettes. In an *Acidovorax* sp. isolate, *bla*_IMP-27_ and *sat*2 gene cassettes were found associated with an insertion sequence, ISL3 transposase, in an RP4 plasmid. The conserved structure of Tn7 in *Shewanella* spp. and *Providencia* spp. is consistent with these species being potential reservoirs from which other bacterial species have acquired partial Tn7 motifs, and the *bla*_IMP-27_ gene cassette. These data contribute to a broader understanding of the dissemination and temporality of *bla*_IMP_ alleles and their mobile genetic elements.

## Introduction

The IMP metallo-beta-lactamase can confer bacterial non-susceptibility or resistance to all beta-lactam antimicrobials, including the carbapenems which are frequently used to treat invasive Gram-negative infections. IMP-producing bacteria are most frequently reported from the South Pacific and Asia [1] and are present but less common in other regions of the world [2]. The *bla*_IMP-1_ allele was first reported in Japan in 1994 as a gene cassette in a class 1 integron [3]. Also found in Japan, the *bla*_IMP-6_ allele has been linked to human infections and hospital outbreaks [4–6]. Elsewhere in the South Pacific, reports from Australia and the Philippines have noted bacterial strains carrying *bla*_IMP-4_ and *bla*_IMP-26_ respectively [1]. Phylogenetic analysis of *bla*_IMP_ alleles shows that *bla*_IMP-6_-related clades share phenotypic characteristics due to a Gly262Ser substitution compared to *bla*_IMP-1_ [7]. Although *bla*_IMP-6_ is found in class 1 integrons, *bla*_IMP-27_, *bla*_IMP-64_, and *bla*_IMP-95_ that we have recovered in North America are almost exclusively found in class 2 integrons. Currently, these alleles have not been linked to human outbreaks or endemic bacterial strains and are recovered mostly commonly from One Health sources [8,9].

Class 2 integrons are non-mobile, with a truncated integrase gene, *intI2*, but maintain their promotor for gene cassette transcription. Class 2 integrons are frequently mobilized in Tn7 transposons [10]. Transposons play an important role in the spread of antimicrobial resistance in that they mobilize genetic elements, including antibiotic resistance genes, to allow their exchange between chromosome and plasmid [11]. The “cut and paste” ability of the Tn7 transposons provides the mechanism for the translocation [12,13] of chromosomally encoded class 2 integrons harboring the *bla*_IMP_ gene cassette to plasmids where they can be transferred horizontally within and between diverse bacterial species. We have observed *bla*_IMP_ within a novel class 2 integron which also harbors *sat2* encoding streptothricin resistance. We found this class 2 integron co-harboring *bla*_IMP_ and *sat2* gene cassettes in multiple taxonomic families of bacteria, both chromosomally and on mobile plasmids, suggesting the temporality of horizontal gene transfer (HGT).

The objective of this study is to characterize the genetic context of *bla*_IMP-27_, *bla*_IMP-64_, *bla*_IMP-95_ alleles in isolates from the *Comamonadaceae*, *Enterobacteriaceae, Moraxellaceae*, *Morganellaceae*, and *Shewenallaceae* families recovered from One Health sources. Understanding the genotypic presentation is important for understanding these genes’ dissemination in a One Health context. This information may aid in our understanding of the temporality of horizontal gene transfer of the class 2 integron that harbors specific *bla*_IMP-27_, *bla*_IMP-64_, and *bla*_IMP-95_ gene cassette alleles.

## Materials and methods

Bacterial isolates harboring the carbapenemase genes, *bla*_IMP-27_, *bla*_IMP-64_, or *bla*_IMP-95_, were originally recovered in Ohio and Missouri between 2015 and 2021 from various One Health sources including wastewater treatment plant effluent, livestock markets, fecal or lagoon samples from dairy and swine, and swabs from animal housing environments. Sampling plans were utilized at each site to ensure that individual samples each represented unique locations within a site. Permits were not required for sampling at these sites, access was provided voluntarily by the facility manager.

For recovery of carbapenem resistant Enterobacterales from samples, we used selective media supplemented with 0.5 μg/mL meropenem and 70 μg/mL zinc sulfate to identify phenotypic carbapenem resistance as previously described [9,14–16]. Carbapenemase production was confirmed in resistant isolates using the CarbaNP test [17], Bacterial species were initially identified by MALDI-TOF (Bruker Scientific, LLC, Billerica, MA), and species not expected to have intrinsic carbapenemase production were tested for the presence of known carbapenemase genes including *bla*_IMP_ by PCR as previously described [9,14–16].

Antimicrobial susceptibility phenotype of the isolates was determined using broth microdilution (Sensititre MIC panels CMV3AGNF or CMV4AGNF and GNX2F, Thermo Fisher Scientific, Oakwood Village, OH) following Clinical and Laboratory Standards Institute (CLSI) guidelines [18]. Whole genome sequencing of the isolates was performed using the Illumina MiSeq platform. Sequence data were quality-checked, adapters removed, then assembled for analysis using SPAdes [19].

Sequences were aligned to a complete Tn7 reference that begins and ends at terminal repeats (IR) of Tn7 (not shown, repeat regions of MN628641.1). Isolates that did not show any evidence of Tn7 in chromosomal contiguous sequences and were found on plasmids were then aligned with an IncQ1 plasmid and original reference KY126032, known to harbor the integron, In2–69. Sequence data were deposited in NCBI GenBank under the Bioproject number PRJNA1197433.

Initial antimicrobial gene identification was accomplished using the ARGAnnot, ResFinder, CARD ontology, and AMRfinder online databases [20–23]. IncQ1 plasmids were confirmed using PlasmidFinder [24]. The ISFinder database [25] was used to identify HGT motifs with initial identification of transposon, insertion sequence, and integrons. For exploration of other truncated and incomplete genes the conserved domain database was used for identifying regions for targeted BLAST searches. Reference genes were then identified using refSeq, available from NCBI GenBank [26]. As well, other annotation tools available in Proksee [27] were used for additional gene annotation [28–32].

## Results

**Fig 1:**
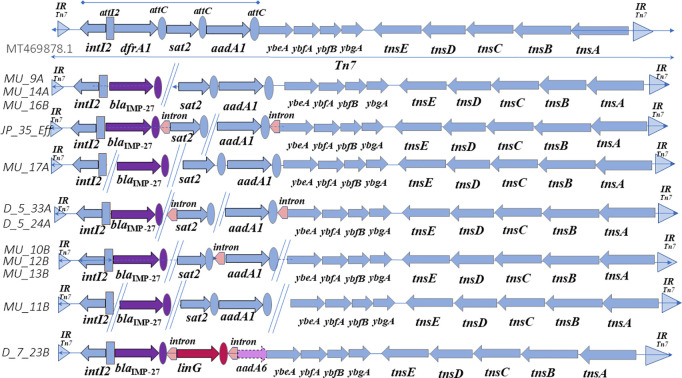
Annotation and alignment of the Tn7 transposon from 12 *Shewanella* spp. isolates harboring *bla*_IMP-27_ recovered from One Health sources to the Tn7 reference MT469878.1 In Fig 1 reference genes are represented by light blue arrows, and arrow direction indicates direction of transcription. Triangles flanking the Tn7 motif represent the attachment site and inverted repeats of *attTn7* (IR). Ovals represent gene cassette recombination sites, and purple shaded elements represent genes not found in the reference, but throughout our samples the carbapenemase producing, *bla*_IMP_. Dashed lines surrounding elements indicate truncations. When not found within contiguous sequence, double lines represent this break at the end of contigs.

**Fig 2:**
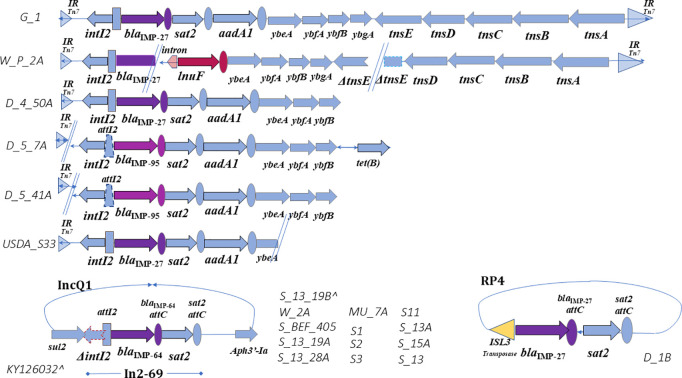
Annotation and alignment of the Tn7 transposon from 2 *Providencia* spp. isolates harboring *bla*_IMP-27_ recovered from One Health sources to the Tn7 reference MT469878.1, and 4 *Acinetobacter* spp. with gene map characterizations with class 2 integrons and alignment of IncQ1 plasmids to integron In2-69, and an RP4 plasmid map In Fig 2 reference genes are represented by light blue arrows, and arrow direction indicates direction of transcription. Triangles flanking the Tn7 motif represent the attachment site and inverted repeats of *attTn7* (IR). Ovals represent gene cassette recombination sites, and purple shaded elements represent genes not found in the reference, but throughout our samples the carbapenemase producing, *bla*_IMP_. Dashed lines surrounding elements indicate truncations. When not found within contiguous sequence, double lines represent this break at the end of contigs.

This study analyzed 32 bacterial isolates, including 12 *Shewanella* spp., 2 *Providencia* spp., 4 *Acinetobacter* spp., 3 *Proteus* spp., 7 *Escherichia coli,* 3 classified only as *Enterobacteriaceae bacterium*, and 1 *Acidovorax* sp. (Table 1) as identified by NCBI. Of these, 17 carried *bla*_IMP-27_, 13 carried *bla*_IMP-64_, and 2 carried *bla*_IMP-95_ (Table 1). Most of the isolates were originally recovered from feces or housing environments of dairy cattle (n = 18) or swine (n = 11), but two were from livestock auction market environments and one from treated human wastewater effluent. In most of the isolates (n = 14), *bla*_IMP-27_ was located within a complete Tn7 transposon (Figs 1, 2). While truncated Tn7 motifs contained *bla*_IMP-27_ (n = 2) and *bla*_IMP-95_ (n = 2), and another 13 isolates maintained *bla*_IMP-64_ on an IncQ1 plasmid and in 1 isolate *bla*_IMP-27_ was located on an RP4 plasmid (Table 1, Fig 2).

**Table 1:** Taxonomic family, *bla*_IMP_ gene index, mechanism for horizontal gene transfer, isolate source, date and US state of collection, and NCBI Genbank SRA accession number for 32 bacterial isolates harboring *bla*_IMP_ obtained from One Health sources.

Isolate	Taxonomy	bla_IMP_ allele^a^	HGT^b^	Source	Collection date	US State	Accession
**JP_35A_EffB**	*Shewanella* sp.	*bla*_IMP-27_	Tn7	Wastewater	06-2017	Ohio	SRR31736511
**D_5_33A**	*Shewanella* sp.	*bla*_IMP-27_	Tn7	Dairy	09-2017	Ohio	SRR31736510
**D_5_24A**	*Shewanella* sp.	*bla*_IMP-27_	Tn7	Dairy	09-2017	Ohio	SRR31736499
**D_7_23B**	*Shewanella* sp.	*bla*_IMP-27_	Tn7	Dairy	09-2017	Ohio	SRR31736488
**MU_9A**	*Shewanella* sp.	*bla*_IMP-27_	Tn7	Dairy	07-2018	Missouri	SRR31736485
**MU_10B**	*Shewanella* sp.	*bla*_IMP-27_	Tn7	Dairy	07-2018	Missouri	SRR31736484
**MU_11B**	*Shewanella* sp.	*bla*_IMP-27_	Tn7	Dairy	07-2018	Missouri	SRR31736483
**MU_12B**	*Shewanella* sp.	*bla*_IMP-27_	Tn7	Dairy	07-2018	Missouri	SRR31736482
**MU_13B**	*Shewanella* sp.	*bla*_IMP-27_	Tn7	Dairy	07-2018	Missouri	SRR31736481
**MU_14A**	*Shewanella* sp.	*bla*_IMP-27_	Tn7	Dairy	08-2018	Missouri	SRR31736480
**MU_17A**	*Shewanella* sp.	*bla*_IMP-27_	Tn7	Dairy	08-2018	Missouri	SRR31736509
**MU_16B**	*Shewanella* sp.	*bla*_IMP-27_	Tn7	Dairy	08-2018	Missouri	SRR31736508
**W_P_2A**	*Providencia* sp.	*bla*_IMP-27_	Tn7	Swine	11-2018	Ohio	SRR31736507
**G_1**	*Providencia* sp.	*bla*_IMP-27_	Tn7	Dairy	02-2018	Ohio	SRR31736506
**D_4_50A**	*Acinetobacter* sp.	*bla*_IMP-27_	ΔTn7	Dairy	12-2016	Ohio	SRR31736505
**USDA_S33**	*Acinetobacter* sp.	*bla*_IMP-27_	ΔTn7	Market	08-2021	Ohio	SRR31736504
**D_5_7A**	*Acinetobacter* sp.	*bla*_IMP-95_	ΔTn7	Dairy	12-2016	Ohio	SRR31736503
**D_5_41A**	*Acinetobacter* sp.	*bla*_IMP-95_	ΔTn7	Dairy	12-2016	Ohio	SRR31736502
**S_13_19B**	*Proteus* sp.	*bla*_IMP-64_	IncQ1	Swine	07-2015	Ohio	SRR31736501
**W_2A**	*Proteus* sp.	*bla*_IMP-64_	IncQ1	Dairy	11-2018	Ohio	SRR31736500
**S_BEF_405**	*Proteus* sp.	*bla*_IMP-64_	IncQ1	Market	07-2017	Ohio	SRR31736498
**S_13_19A**	*E. coli*	*bla*_IMP-64_	IncQ1	Swine	07-2015	Ohio	SRR31736497
**S_13_28A**	*E. coli*	*bla*_IMP-64_	IncQ1	Swine	07-2015	Ohio	SRR31736496
**MU_7A_1**	*E. coli*	*bla*_IMP-64_	IncQ1	Dairy	07-2018	Missouri	SRR31736495
**S1**	*E. coli*	*bla*_IMP-64_	IncQ1	Swine	05-2016	Ohio	SRR31736494
**S2**	*E. coli*	*bla*_IMP-64_	IncQ1	Swine	05-2016	Ohio	SRR31736493
**S3**	*E. coli*	*bla*_IMP-64_	IncQ1	Swine	05-2016	Ohio	SRR31736492
**S11**	*E. coli*	*bla*_IMP-64_	IncQ1	Swine	05-2016	Ohio	SRR31736491
**S_13A**	*Enterobacteriaceae bacterium*	*bla*_IMP-64_	IncQ1	Swine	05-2016	Ohio	SRR31736490
**S_15A**	*Enterobacteriaceae bacterium*	*bla*_IMP-64_	IncQ1	Swine	05-2016	Ohio	SRR31736489
**S_13**	*Enterobacteriaceae bacterium*	*bla*_IMP-64_	IncQ1	Swine	05-2016	Ohio	SRR31736487
**D_1B**	*Acidovorax* sp.	*bla*_IMP-27_	RP4	Dairy	09-2017	Ohio	SRR31736486

^a^*bla*_IMP_ consists of 741 bp of which there is a single nucleotide difference between *bla*_IMP-27_ and *bla*_IMP-64_. *bla*_IMP-95_ has 18 nucleotide differences compared to *bla*_IMP-27_ and 19 nucleotide differences compare to *bla*_IMP-64_.

^b^Horizontal gene transfer (HGT) is either transposon-mediated (Tn7) or plasmid-mediated (IncQ1or RP4). ΔTn7 indicates a truncated Tn7 transposon motif missing genomic evidence of a functional transposition element.

We observed taxon-specific differences in the genetic context of *bla*_IMP_ gene alleles. *Shewanella* spp. and *Providencia* spp. isolates only contained *bla*_IMP-27_ within a complete Tn7. Among multiple *Shewanella* spp. and *Providencia* spp. in our study, the presence of the Tn7 transposon, with the class 2 integron containing the *bla*_IMP-27_ gene cassette, was located adjacent to the *glmS* gene (glucosamine 6-phosphate synthase). Eleven of the *Shewanella* spp. isolates and both of the *Providencia* spp. contain Tn7 just upstream from *glmS,* a known Tn7 attachment site, *attTn7* [33]. Only one *Shewanella* sp. isolate (MU_12B) did not show this *attTn7* to be present near *glmS* (Figs S1–S5). All *Shewanella* spp. isolates, except MU_12B, had complete Tn7s as well as the specific attachment site, *attTn7*, downstream of *glmS* as shown in annotation of the contiguous sequence from *Shewanella* sp. isolate D_7_23B (Fig S3). This is the most common and known attachment site for Tn7 within bacteria [33,34]. This is also seen in a contiguous sequence of *Providencia* sp. isolate G_1, that shows the complete Tn7 adjacent to the known *glmS* attachment site. All but one *Shewanella* spp., isolate MU_12B, show a transposition event to the *glmS* attachment site. In chromosomal sequence, the Tn7 flanking inverted, direct repeats (IR) at attachment sites (Figs 1, S1–S3, S6) provide evidence that *Shewanella* spp. and *Providencia* spp. isolates acquired *bla*_IMP-27_ from a transposition event.

In MU_12B, multiple upstream elements atypical for a Tn7 attachment site were observed (Fig S4). Where *glmS* is found in MU_12B there is no sign of a Tn7 element, and where the Tn7 is located, there are a number of other functional mechanisms (Figs S4, S5). MU_12B also shows the Tn7 right end 22 bp repeat region adjacent to the Tn7 motif and the Tn7 left end 22 bp repeat region adjacent to *intI2* (Figs S1, S2, S4, S6). The three gene cassettes for the class 2 integron in MU_12B are found on two different contigs (S6, S7). In addition to *bla*_IMP-27_, *Shewanella* spp. isolates also harbored an additional carbapenemase gene *bla*_OXA-48_ or *bla*_OXA-48-like_ on distant contigs (data not shown).

In contrast, *Acinetobacter* spp. isolates show evidence of prior acquisition, by harboring *bla*_IMP-27_ or *bla*_IMP-95_ within truncated Tn7 motifs in chromosomally associated contiguous sequences, allowing continual vertical transmission, without a fully functional Tn7 transposition mechanism surrounding the class 2 integron (Fig 2). Without the genes required for a transposition event (*tns*ABCDE), there is no genomic evidence of a functional transposition element (Fig 2, isolates D_4_50A, D_5_7A, D_5_41A, and USDA_S33). In these isolates, the Tn7 gene array was not found beyond what is described in the gene maps (Fig 2).

*Proteus* spp., *E. coli*, and the *Enterobacteriaceae bacterium* isolates show that the IncQ1 plasmid retains the class 2 integron motif but does not have evidence of a Tn7 transposition event as no evidence of any Tn7 motifs were found within chromosomal or other plasmid contigs for these isolates (Fig 2).

## Discussion

The genetic context of *bla*_IMP_ varied by taxonomic family, reflecting different HGT mechanisms. *Shewanella* spp. and *Providencia* spp. isolates all maintained *bla*_IMP-27_ within a complete chromosomal Tn7 transposon while *Acinetobacter* spp. maintained *bla*_IMP-27_ and *bla*_IMP-95_ within a truncated, incomplete chromosomal Tn7. Other *Enterobacteriaceae* including *Proteus* spp., *E. coli*, and the isolates classified by NCBI as *Enterobacteriaceae bacterium* harbored *bla*_IMP-64_ on an IncQ1 plasmid, and a single *Acidovorax* sp. isolate harbored *bla*_IMP-27_ and *sat*2 gene cassettes on a RP4 plasmid, absent association to a class 2 integron, and adjacent to an ISL3. This suggests that taxonomy may influence both the specific *bla*_IMP_ allele present and the predominant HGT mechanism, whether via transposition or plasmid exchange.

*Shewanella* spp. and *Providencia* spp. isolates are the only taxonomic families where the complete Tn7 was found and only harbor the *bla*_IMP-27_ gene allele. *Acinetobacter* spp. isolates were found with incomplete Tn7 chromosomally, missing the functional transposition elements (*tns*ABCDE), but with evidence of a prior transposition event due to the IR of *att*Tn7 found up stream of the class 2 integron, and with no other functional Tn7 elements found in other contiguous sequences in these isolates (Fig 2). *Acinetobacter* spp. isolates harbor both *bla*_IMP-27_ and *bla*_IMP-95_. Curiously, all *bla*_IMP-64_ genes were only found in IncQ1 plasmids present in *Proteus* spp., *E. coli,* and the isolates identified by NCBI only as *Enterobacteriaceae bacterium*. A single RP4 plasmid within an *Acidovorax* sp. isolate harbors a *bla*_IMP-27_ and *sat*2 but is absent of the class2 integron, with a ISL3 transposase in its place.

Tn7 is a transposon known for the ability to “cut and paste” into a specific chromosomal target site. TnsABC + TnsD within Tn7 control and direct transposition to the *attTn7*, directly downstream of *glmS* [33,34]. This site-specific transposition location allows incorporation into the host bacteria, without the inactivation of any of the host bacteria’s genetic mechanisms [34]. Tn7 also has target site immunity, not allowing multiple copies of Tn7 to exist within the same host [34]. TnsABC + TnsE is the mechanism by which the Tn7 is directed into conjugative plasmids [11]. A single mutation in *tns*A can induce extensive DNA replication, converting Tn7 into a replicative transposon [35], thus potentially increasing gene cassette copy number.

Tn7 transposons have long been identified as carriers of class 2 integrons [36], so it is unsurprising to note the class 2 integron containing a *bla*_IMP_ gene cassette within a Tn7, as is the case with all *Shewanella* spp. and *Providencia* spp. isolates. The functionality of the Tn7 and the ability to cut and paste from chromosome to plasmid, and then plasmid to chromosome, gives the ability of resistance gene cassettes to be found across diverse bacterial species [12,13]. When the class 2 integron is found within the Tn7, any associated HGT is the result of a transposition event.

Complete Tn7s found in our isolates differ from Tn7 alignment references (MT469878.1 in Figs 1 and 2, and MN62864.1 in Figs S1–S3) in that the first gene cassette found in class 2 integrons, typically *dfr*A1 (trimethoprim resistance) has been replaced by a *bla*_IMP_ (carbapenem resistance) (Fig 1). In the typical 2^nd^ and 3^rd^ gene cassettes, found in most isolates are genes for *sat*2 and *aad*A1 (resistance to streptothricin and streptomycin/ spectinomycin, respectively). The exceptions for the 2^nd^ gene cassette being in D_7_23B and W_P_2A where the typical *sat*2 gene has been substituted by *lin*G and *lnu*F respectively (Figs 1 and 2). In isolates where the *bla*_IMP_ and *sat*2 gene cassettes are found in plasmids, the typical *aad*A genes in the 3^rd^ cassette position are absent (Fig 2).

*Morganellaceae* harboring *bla*_IMP-27_ within a Tn7 transposon were previously reported for 23 Canadian isolates identified from routine surveillance for carbapenemase production among Gram-negative bacteria [37]. Curiously, they did not note the second gene cassette for *sat2* that we observed being present in the resistome, with the caveat that *sat2* is not in the ResFinder database and so may not have been detected. We have found that ResFinder consistently fails to detect *sat*2, emphasizing the need to consult multiple resistant gene annotation tools. We have found that using multiple annotation tools including ARGAnnot and CARD provide further confirmation of the correct annotation for the *sat*2. In other GenBank references, we have found the *bla*_IMP-27_-*sat*2-*aad*A1 gene cassette motif to be consistently present (as noted in Boyd, 2021 Fig 1) [37–39]. Less common Enterobacterales, such as *Providencia* spp. and *Proteus* sp., have been previously noted to harbor *bla*_IMP_ [40]. Class 1 and class 3 integrons are established carriers of major *bla*_IMP_ alleles [41]. Alleles of *bla*_IMP_ and their distribution in bacterial species have been described [42]. A study where eleven isolates from American crows were found to carry two similar Tn7 genetic clusters to one of those within our study (W_P_2A), with the same *bla*_IMP-27_ motif in a Tn7, on chromosomes of *Providencia rettgeri*, with a similar substitution in the second gene cassette, of an *lnu*(f)-like rather than a *sat*2 gene [8]. The *bla*_IMP-27_ has also been previously found on transferable plasmids in *Providencia* spp. and *Proteus* spp. but without the plasmid replicon type identified. [43].

However, when a truncated Tn7 contains a class 2 integron, we can only speculate as to how the vertical transmission of the resistance gene motif is maintained and when and whether further mutations allow this element to be further spread horizontally. It is perhaps possible that the integron was acquired through transformation in the *Acinetobacter* spp., as there are no plasmids, or complete Tn7 motifs present, and 3 of the 4 isolates are predictive for CRISPR-Cas system [44].We first identified the IncQ1 plasmid harboring a *bla*_IMP-64_ gene cassette in a class 2 integron [16]. IncQ plasmids are small, non-conjugative, and self-replicating. Although non-conjugative, they are efficiently transferred by a mobilization process which is attributable to other conjugative, helper plasmids, or by parasitizing of type IV secretion systems [45]. The presence of complete class 2 integrons on IncQ1 plasmids, absent evidence of transposition, suggests prior acquisition via transposition or transformation in an ancestral bacterial lineage, followed by stable maintenance through mutation. The IncQ plasmid is of importance in a One Health context as it has a very broad host range, notably it has been identified in wastewater as an important reservoir for antimicrobial resistance genes [46].

It is unclear how a RP4 plasmid picked up the two resistant gene cassettes *bla*_IMP-27_-*sat*2 that are typically associated with a class 2 integron. The functionality of the resistance genes is determined by the promoter region of the integrase, and in this case, would therefore maintain the carbapenemase functionality from a promoter region within the ISL3. As all isolates were identified as carbapenemase producers, and no other genomic analysis shows any other carbapenem resistance genes present within the *Acidovorax* sp. isolate, it is evident that the *bla*_IMP-27_ is functional.

As has been noted in other studies, the prevalence of certain *bla*_IMP_ alleles may be underestimated and unexamined depending on what carbapenemase gene PCR primers are used, laboratory assays and carbapenemase production confirmation analysis [37–39]. Our results may be limited because of the use of short read sequencing, where genetic motifs are determined based on assemblies and alignment when Tn7, integrons, and resistance genes are found in different contigs. Had we been able to utilize long-read sequencing, gene location would have been more precise. It is also possible that some mobile elements were not recognized within certain bacterial species. PlasmidFinder did not identify the RP4 plasmid, although both SPAdes (with plasmid specification) [19] and Galaxy [47] gave circularized closed assemblies, and careful annotation identified the origin of transfer, and known RP4 *tra*J and *tra*J-II genes. It is possible that although PlasmidFinder did not identify any plasmids within the *Shewanella* spp. and *Providencia* spp., and there were no obvious plasmids on assembly, that mechanisms could exist for the Tn7 to become mobilized to a plasmid if we could better identify certain plasmid motifs. As bioinformatic tools improve, it is likely that isolates like these obtained from One Health sources may further help us understand the temporality and spread of the *bla*_IMP-27_ *bla*_IMP-64_ and *bla*_IMP-95_ gene alleles.

## Supporting information

S1 Fig Alignment to transposon Tn7 right end, evidence of four 22 bp repeats, the TnsB binding site as exampled in *Shewanella* sp. isolate MU_12B harboring *bla*_IMP-27_. (TIF)

S2 Fig Alignment to transposon Tn7 left end, evidence of 3 22 bp repeats, and the TnsB binding site as exampled in *Shewanella* sp. isolate MU_12B harboring *bla*_IMP-27_. (TIF)

S3 Fig Complete transposon Tn7 adjacent to *glmS* in *Shewanella* sp. isolate D_7_23B harboring *bla*_IMP-27_ as mapped and annotated in Proksee. (TIF)

S4 Fig Tn7 region absent *glmS* gene in *Shewanella* sp. MU_12B harboring *bla*_IMP-27_ as mapped and annotated in Proksee.
(TIF)

S5 Fig Region with *glmS* gene absent Tn7 in *Shewanella* sp. MU_12B harboring *bla*_IMP-27_ as mapped and annotated in Proksee.
(TIF)

S6 Fig Resistance gene, *bla*_IMP-27_, *intI2,* and Tn7L region in *Shewanella* sp. MU_12B harboring *bla*_IMP-27_ as mapped and annotated in Proksee.
(TIF)

S7 Fig Resistance genes *sat2*, type ii intron, and *aadA1* in *Shewanella* sp. MU_12B harboring *bla*_IMP-27_ as mapped and annotated in Proksee.
(TIF)
