# Supplementary figures and images for: The genetic context of blaIMP varies among bacterial families from One Health sources
Source: PLoS One. 2025 Jul 23;20(7):e0327200. doi: 10.1371/journal.pone.0327200 (PMC12286353; doi:10.1371/journal.pone.0327200)

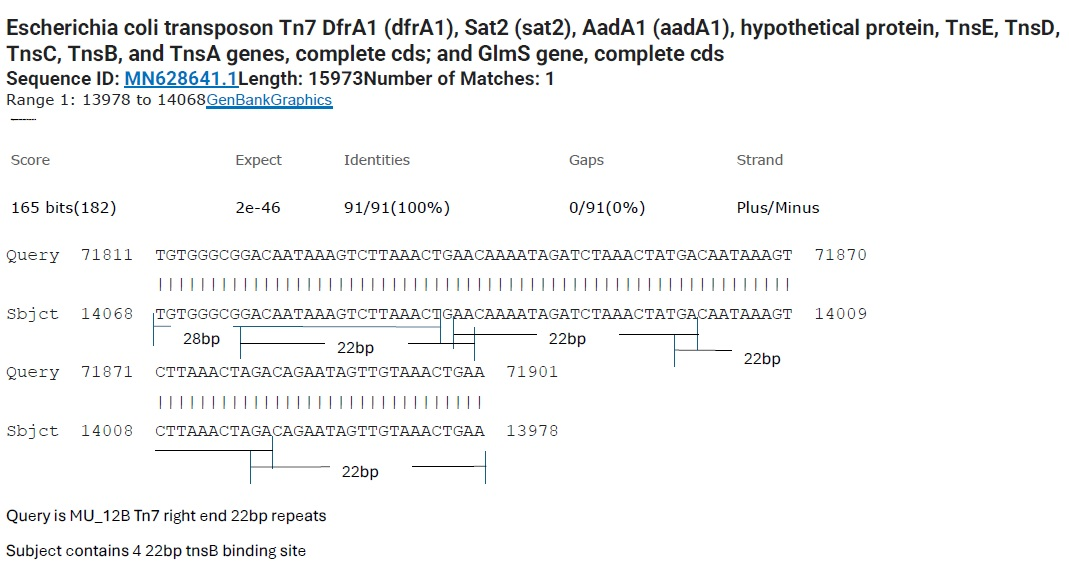

Supplement: S1 Fig — (TIF) [file pone.0327200.s001.tif]

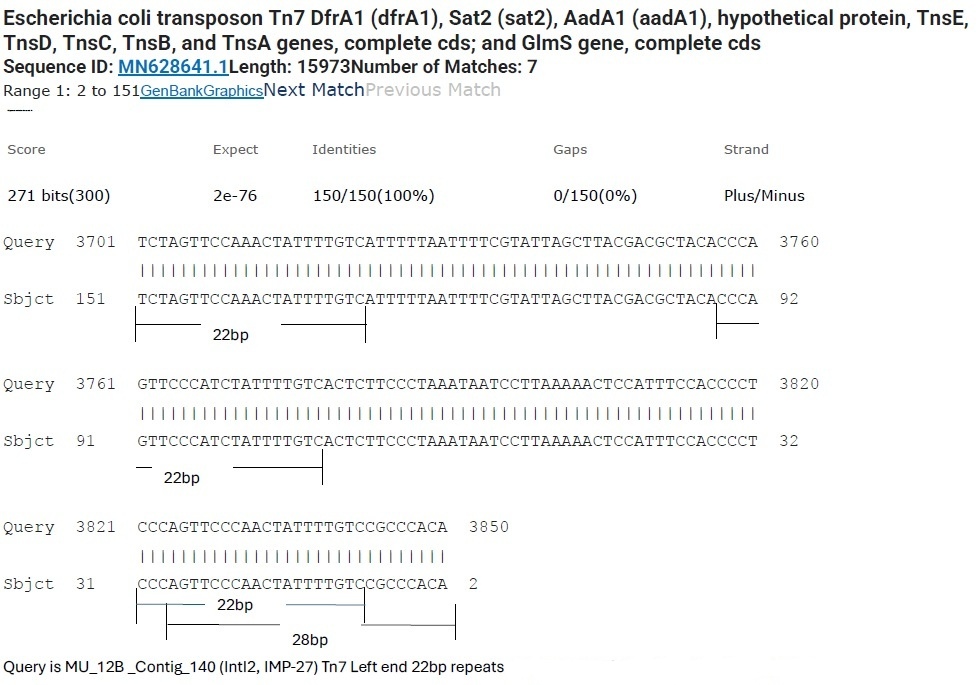

Supplement: S2 Fig — (TIF) [file pone.0327200.s002.tif]

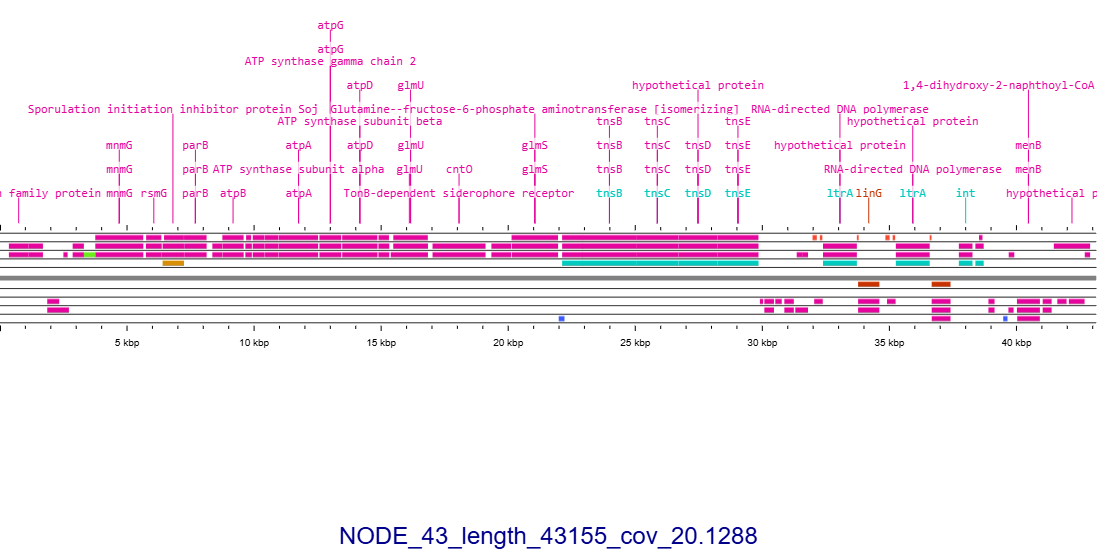

Supplement: S3 Fig — (TIF) [file pone.0327200.s003.tif]

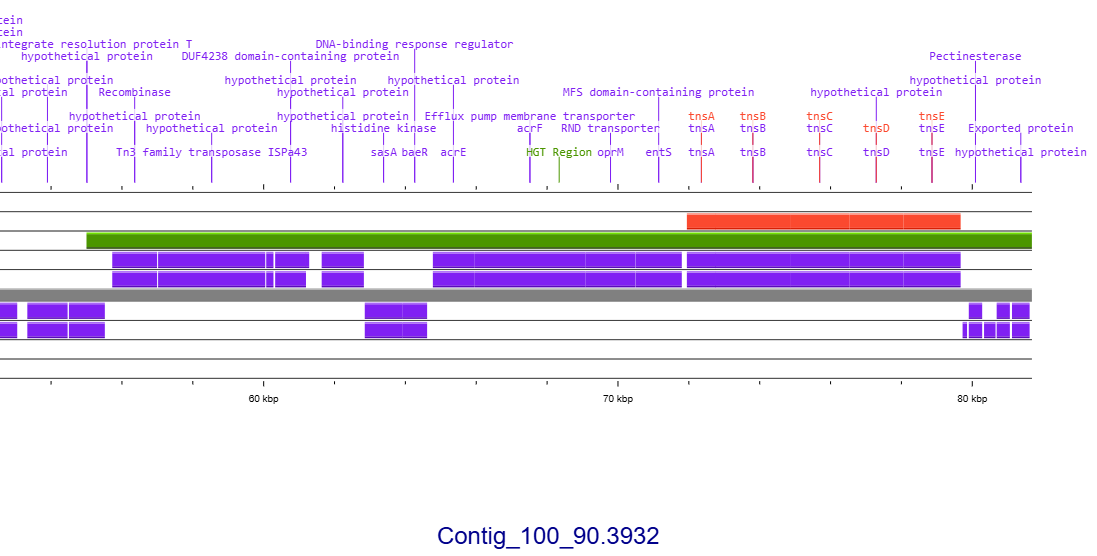

Supplement: S4 Fig — MU_12B harboring blaIMP-27 as mapped and annotated in Proksee. (TIF) [file pone.0327200.s004.tif]

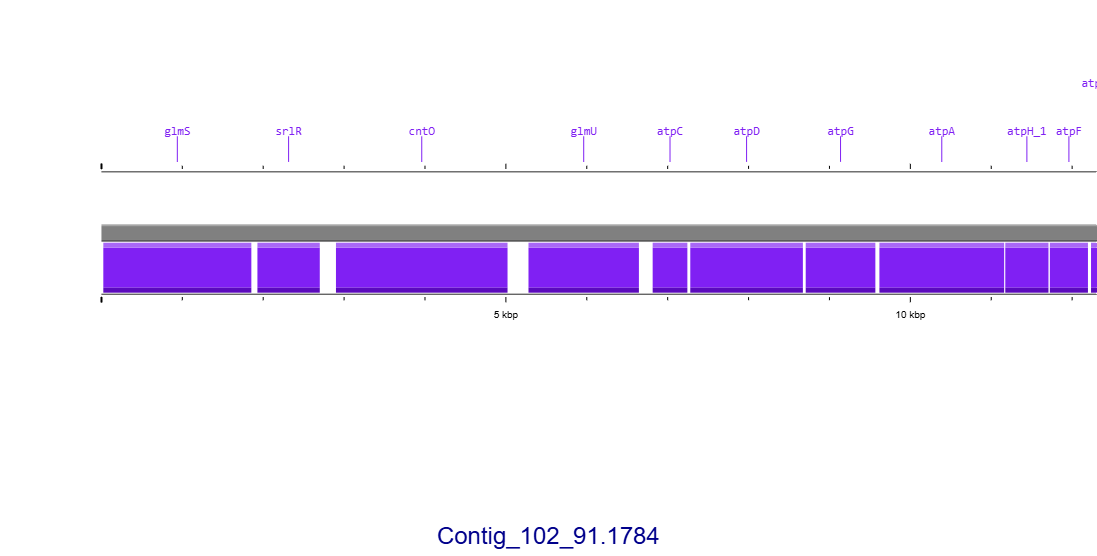

Supplement: S5 Fig — MU_12B harboring blaIMP-27 as mapped and annotated in Proksee. (TIF) [file pone.0327200.s005.tif]

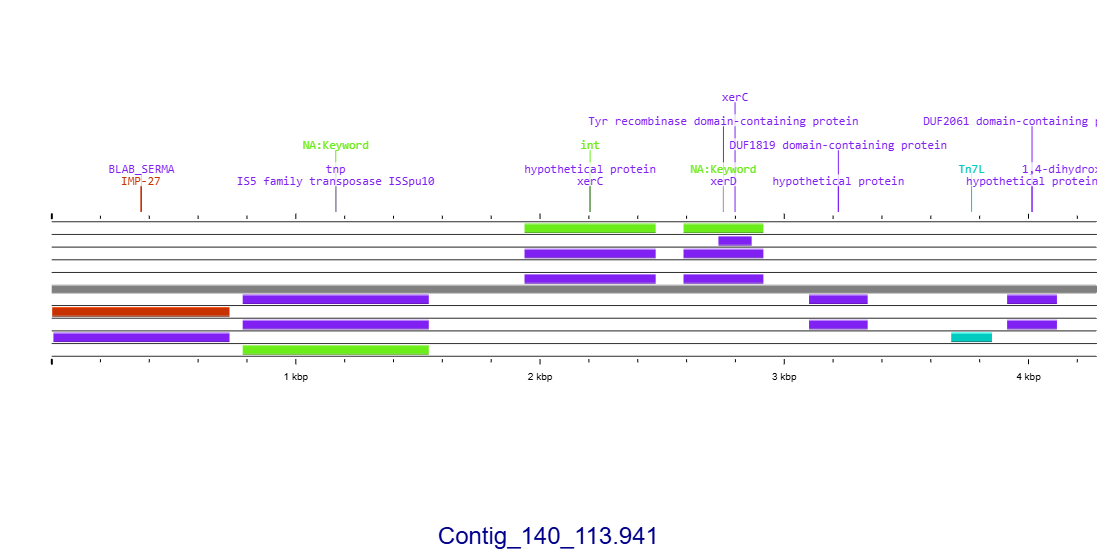

Supplement: S6 Fig — MU_12B harboring blaIMP-27 as mapped and annotated in Proksee. (TIF) [file pone.0327200.s006.tif]

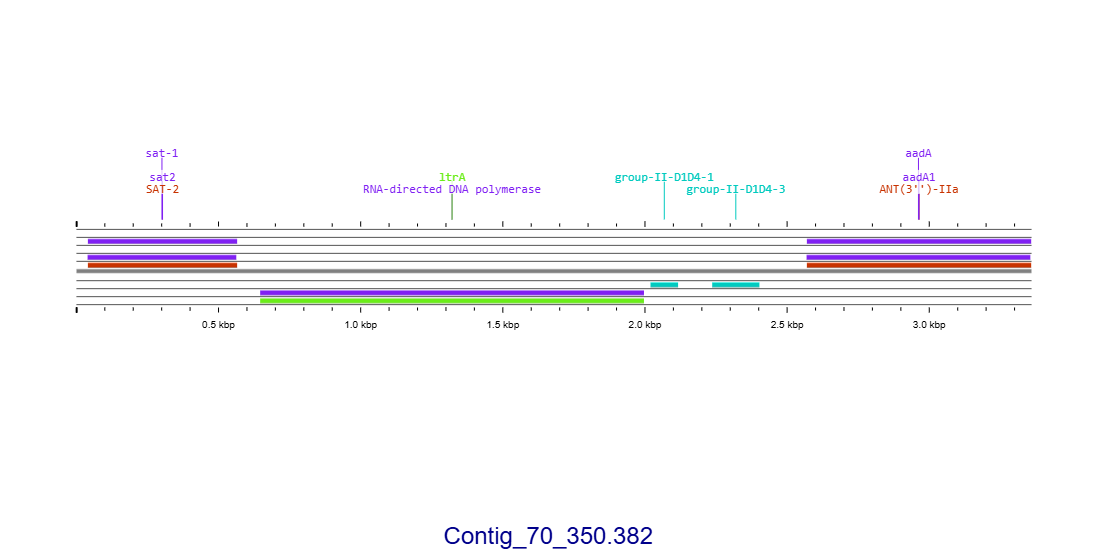

Supplement: S7 Fig — MU_12B harboring blaIMP-27 as mapped and annotated in Proksee. (TIF) [file pone.0327200.s007.tif]
